# Moxibustion for treating knee osteoarthritis: study protocol of a multicentre randomised controlled trial

**DOI:** 10.1186/1472-6882-13-59

**Published:** 2013-03-13

**Authors:** Seunghoon Lee, Kun Hyung Kim, Tae-Hun Kim, Jung-Eun Kim, Joo-Hee Kim, Jung Won Kang, Kyung-Won Kang, So-Young Jung, Ae-Ran Kim, Hyo-Ju Park, Mi-Suk Shin, Kwon-Eui Hong, Ho-Sueb Song, Jin-Bong Choi, Hyung-Jun Kim, Sun-Mi Choi

**Affiliations:** 1 Acupuncture, Moxibustion & Meridian Research Group, Medical Research Division, Korea Institute of Oriental Medicine, Daejeon, South Korea; 2 Department of Acupuncture & Moxibustion, College of Korean Medicine, Kyung Hee University, Seoul, South Korea; 3 Department of Acupuncture & Moxibustion Medicine, Korean Medicine Hospital, Pusan National University, Pusan, South Korea; 4 Mokhuri Neck and Back Hospital, Seoul, South Korea; 5 Department of Acupuncture & Moxibustion, College of Oriental Medicine, Daejeon University, Daejeon, South Korea; 6 Department of Acupuncture & Moxibustion, College of Oriental Medicine, Gachon University, Incheon, South Korea; 7 Department of Oriental Rehabilitation Medicine, College of Oriental Medicine, Dong-Shin University, Gwangju, South Korea; 8 Department of Oriental Gynaecology, College of Oriental Medicine, Se-Myung University, Jecheon, South Korea

## Abstract

**Background:**

The treatment of knee osteoarthritis, which is a major cause of disability among the elderly, is typically selected from multidisciplinary options, including complementary and alternative medicine. Moxibustion has been used in the treatment of knee osteoarthritis in Korea to reduce pain and improve physical activity. However, there is no sufficient evidence of its effectiveness, and it cannot therefore be widely recommended for treating knee osteoarthritis. We designed a randomised controlled clinical trial to evaluate the effectiveness, safety, cost-effectiveness, and qualitative characteristics of moxibustion treatment of knee osteoarthritis compared to usual care.

**Methods/designs:**

This is a protocol for a multicentre, pragmatic, randomised, assessor-blinded, controlled, parallel-group study. A total of 212 participants will be assigned to the moxibustion group (n = 106) and the usual care group (n = 106) at 4 clinical research centres. The participants assigned to the moxibustion group will receive moxibustion treatment of the affected knee(s) at 6 standard acupuncture points (ST36, ST35, ST34, SP9, Ex-LE04, and SP10) 3 times per week for 4 weeks (a total of 12 sessions). Participants in the usual care group will not receive moxibustion treatment during the study period. Follow-up will be performed on the 5th and 13th weeks after random allocation. Both groups will be allowed to use any type of treatment, including surgery, conventional medication, physical treatment, acupuncture, herbal medicine, over-the-counter drugs, and other active treatments. Educational material that explains knee osteoarthritis, the current management options, and self-exercise will be provided to each group. The global scale of the Korean Western Ontario and McMaster Osteoarthritis Index (K-WOMAC) will be the primary outcome measurement used in this study. Other subscales (pain, stiffness, and function) of the K-WOMAC, the Short-Form 36v2 Health Survey, the Beck Depression Inventory, the Physical Function test, Patient Global Assessment, and the Pain Numerical Rating Scale will be used as outcome variables to evaluate the effectiveness of moxibustion. Safety will be assessed at every visit. In addition, an economic evaluation and a qualitative study will be conducted as a mixed-methods approach.

**Discussion:**

This trial may contribute to developing evidence for the effectiveness and safety of moxibustion for treating knee osteoarthritis.

**Trial registration:**

Trial registration number: KCT0000130

## Background

Osteoarthritis (OA) is the most common form of arthritis in the elderly, and it is characterised by pain and functional limitation, leading to a reduction in quality of life [1,2]. It is an active disease process involving the focal loss of articular cartilage, subchondral bone thickening, and new bone formation [3].

Non-steroidal anti-inflammatory drugs (NSAIDs) and acetaminophen are the most common pharmacological agents used to treat OA. However, according to recent studies, these products only help to slightly reduce short-term pain and do not inhibit disease progression [4,5]. Furthermore, these drugs are associated with adverse events, such as gastrointestinal irritation and bleeding, perforating ulcers, and renal and hepatic toxicity. Therefore, patients who experience unsatisfactory results and a risk of adverse events using pharmacological agents are recommended to pursue non-pharmacological treatments, including complementary and alternative medicines, such as acupuncture and moxibustion [6].

Moxibustion is a traditional oriental therapy that treats diseases through thermal stimulation by burning herbs, primarily *Artemisia vulgaris*, at specific spots on the skin [7]. It has been used for various diseases, such as OA, stroke, and hot flushes, in addition to acupuncture in the traditional medical systems of Korea, China, Japan, Vietnam, and Mongolia [8].

Acupuncture has been studied sufficiently to demonstrate that it has benefits for peripheral knee osteoarthritis (KOA) [6,9]. One systematic review evaluated acupuncture for peripheral joint OA and included 14 randomised controlled trials (RCTs) for KOA (a total of 18 RCTs); acupuncture was shown to be efficacious for pain control in KOA in sham-controlled RCTs [6]. In the Cochrane review, the authors concluded that acupuncture for peripheral joint OA had statistically significant and clinically relevant benefits in waiting list-controlled trials [9].

However, according to a recent systematic review [10] on the use of moxibustion for rheumatic conditions, only 4 RCTs [11-14] on KOA using moxibustion have been published, and it is difficult to provide conclusive evidence for the effectiveness of moxibustion in treating rheumatic conditions, including KOA because of a high risk of bias with low methodological quality. Therefore, high-quality studies following the CONSORT 2010 Statement and revised STRICTA are necessary to determine whether moxibustion is an effective treatment for KOA [15,16]. In addition, even if a placebo moxibustion procedure was developed, a successfully validated placebo moxibustion method that could be used in a double-blind RCT does not yet exist [17,18]. Thus, a pragmatic design using a usual care group is appropriate for evaluating overall effectiveness in a moxibustion trial [19].

More than half of Doctors of Korean Medicine (DKMs) use moxibustion to treat KOA [20], and moxibustion is one of the most common complementary and alternative medicines in Korea [21]. Because of the wide prevalence of moxibustion in Korea, it is necessary to evaluate the economic worth of moxibustion for treating KOA, and a qualitative study is needed in addition to the quantitative approach to understand why KOA patients seek moxibustion treatment and how they regard moxibustion treatment performed by a DKM.

Our study will therefore take a pragmatic approach and use a process of randomisation to assess the effectiveness and safety of moxibustion for treating KOA compared to usual care with a sample size calculated on the basis of a previous pilot study. Moreover, an economic analysis will be evaluated to assess the economic value of moxibustion in Korea, and a qualitative study will be conducted to understand the patient’s experience and perspective on the use of moxibustion to treat KOA.

## Methods/design

### Objective

The aims of this study are as follows: (1) to assess the effectiveness of moxibustion treatment for treating KOA on the occurrence of pain, function, depression, quality of life, and adverse events compared to usual care and (2) to assess the cost-effectiveness and cost-utility of moxibustion in addition to usual care in the treatment of KOA patients and explore how KOA patients experience moxibustion in Korea.

### Design and setting

**Figure 1:**
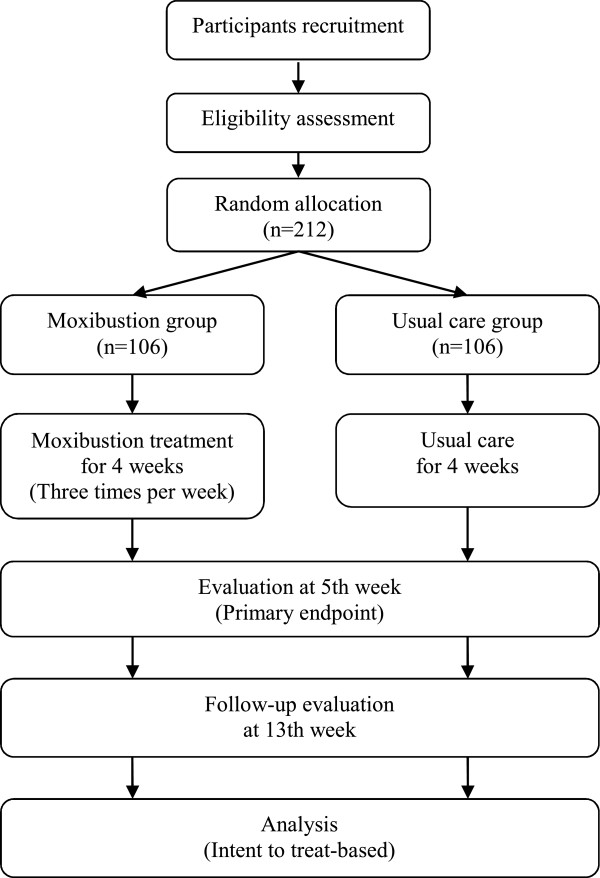
A flowchart of the study process.

This is a multicentre, pragmatic, randomised, assessor-blinded, controlled, parallel-group study. From May 2011 to November 2011, 4 clinical research centres in Korea will participate in this trial: the Korea Institute of Oriental Medicine (Daejeon University Hospital) in Daejeon, Gachon University Gil Oriental Medical Hospital in Incheon, Dongshin University Gwangju Oriental Hospital in Gwangju, and Semyung University Oriental Medicine Hospital in Jecheon. The population of each city is greater than 140,000, and the estimated prevalence rates of KOA are 14.9% over 40 years old [22] and 38.1% over 65 years old [23] in Korea. Recruitment will therefore be expected to be completed within the study period. To recruit the participants, we will advertise in the media (daily papers, local newspapers, and public newsletters), in each hospital, and on the internet homepages of hospitals and public institutions. Eligible participants will be randomly allocated into 2 groups (the moxibustion group or the usual care group) with a 1:1 allocation ratio and receive treatment for 4 weeks with 2 months of follow-up (Figure 1). The evaluation of participants and the analysis of the results will be performed by professionals blinded to the group allocation. Written consent will be obtained from each participant before the start of this trial. We will obtain oral and written consent from each participant before collecting information at the first visit.

### Types of participants

#### Inclusion criteria

A total of 212 participants with KOA will be recruited from the outpatients of the acupuncture and moxibustion clinics of the 4 hospitals. We will include participants who have average daily pain over 40 points (on a 0- to 100-point scale) and have been diagnosed with idiopathic KOA according to the clinical guidelines of the American College of Rheumatology. The guidelines include patients currently experiencing pain in one or both knees with at least 3 of the following 6 conditions: 1) aged 50 to 70 years, 2) morning stiffness within 30 minutes of waking, 3) crepitus, 4) bony tenderness, 5) bony enlargement, and 6) no palpable warmth.

### Exclusion criteria

Participants will be excluded if they are experiencing or have a history of the following: inflammatory diseases, including rheumatoid arthritis; cancer; traumatic injury that might be related to the current knee pain; autoimmune disease; uncontrolled hypertension; diabetes mellitus requiring insulin injection; life-threatening cardiovascular or neurological events within the past year; chronic respiratory disease; a haemorrhagic disorder; alcohol or drug addiction; an active infectious disease, including tuberculosis; keloidosis; a significant knee joint deformity; knee replacement surgery for the affected knee; knee arthroscopy within the past 2 years; steroid injection in the knee joints within the past 3 months; viscosupplement injections in the knee joints within the past 6 months; joint fluid injection within the past 6 months; and acupuncture, moxibustion, cupping, or herbal medicine within the past 4 weeks.

### Randomisation and allocation concealment

Randomisation will be performed using a computerised random number generator through the stratified block randomisation method of the SAS package (Version 9.1.3; SAS institute Inc., Cary, NC, USA) for sequence generation prepared by a statistician (KWK) with no clinical involvement in this trial. According to the Kellgren/Lawrence Scale of anterior-posterior (AP) and lateral standing knee radiographs, a random number will be stratified using a random block size of 4. Sequentially numbered, opaque, sealed assignment envelopes will be delivered to each research centre. Random allocation will be performed at the second visit for participants who provide informed consent and meet the criteria for inclusion. If a participant can be included in the study, the researcher will open the corresponding envelope in front of the participant. The corresponding envelopes will be opened only after the enrolled participants have completed all baseline assessments. Although participants and practitioners will be aware of the allocation arm according to the routine care setting, the outcome assessors and the data analysts will be kept blinded to the allocation.

### Intervention

#### Moxibustion treatment

Moxibustion treatment will be performed by DKMs who are certified by the Korean Ministry of Health and Welfare. They will have at least 2 years of clinical experience and will have received more than 6 years of oriental medicine college education. The treatment will be performed under the supervision of professors who specialise in acupuncture and moxibustion. All treatment regimens and outcome assessment methods will be standardised between the 4 centres, and basic information about this study and the monitoring process will be disseminated through workshops before the beginning of the study. This study is designed to evaluate moxibustion treatment as it is used in normal practice. The practitioner will therefore be allowed to maintain a patient-practitioner relationship similar to a normal clinical situation. There will be no restrictions in the careful observation and questioning of participants.

**Figure 2:**
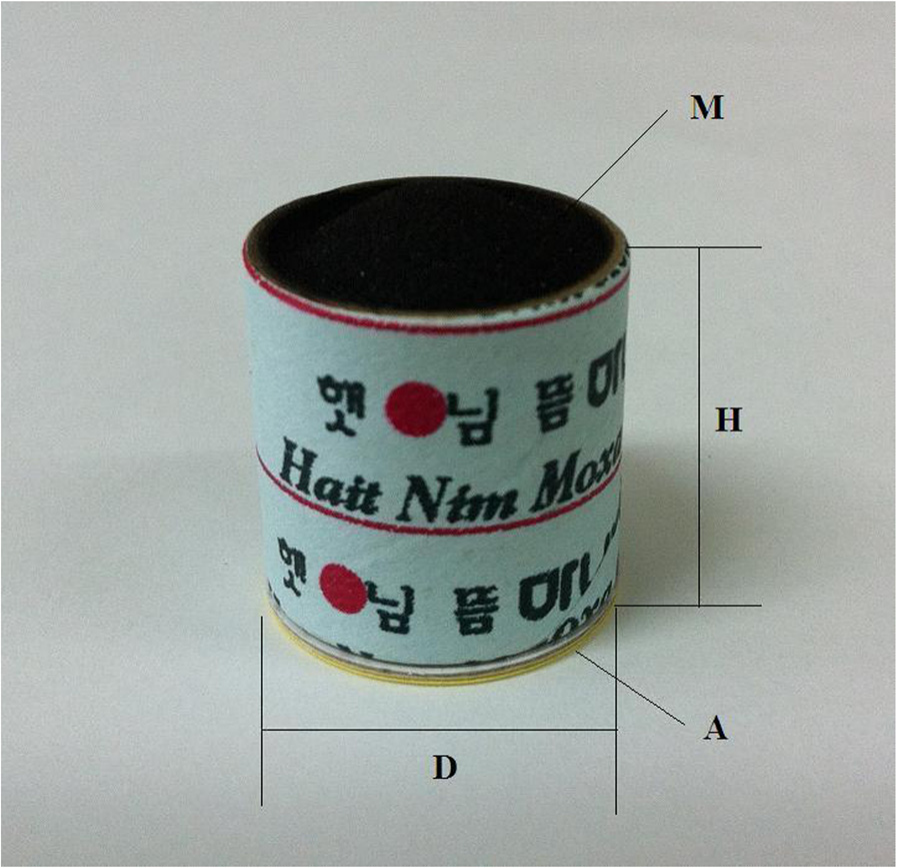
**Moxibustion device.** A photograph of the moxibustion device that will be used in this trial. This moxibustion device is a sticker-type disposable moxa, which functions for 5–10 minutes and is used for local acupuncture points. M: the upper part of the carbonised mugwort, A: adhesive sticker, H: height (2.1 cm), D: distance (1.9 cm).

**Figure 3:**
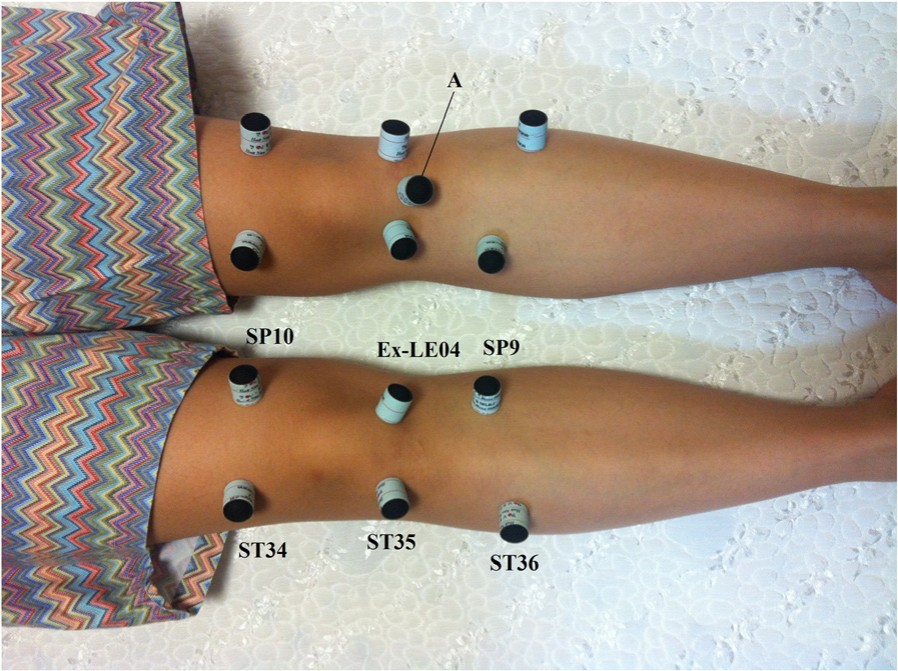
**Acupuncture points of treatment.** The right knee will be treated at 6 standard acupuncture points (ST36, ST35, ST34, SP9, Ex-LE04, and SP10). The left knee will be treated with an additional point of ‘ashi’, which is located between ST35 and Ex-LE04. A: additional points of ‘ashi’ according to the determination of the practitioner. A Written consent for the picture was obtained from the pictured subject. Photograph by Seunghoon Lee.

In the moxibustion group, 3 moxibustion sessions per week will be performed during a 4-week period for a total of 12 sessions. The treatment will be conducted after sterilising the skin surface at the acupuncture points with the participant lying in a supine position at a room temperature of 22°C to 28°C. After the top of the moxibustion is sufficiently combusted without attachment to the points for approximately 5 to 10 seconds, the moxibustion will be attached at each point. When a participant believes that their heat tolerance has reached its maximum, the practitioners will place a new moxibustion at the same point. However, to prevent burns, the attached point can be changed to within a 1 cm diameter according to the discretion of the practitioners. A total of 3 moxibustions will be attached at each point at every visit. We will use smokeless indirect moxibustions made of mugwort with a paper cylinder (Manina, Haitnim Co., Korea) (Figure 2). This moxibustion has an adhesive on the bottom that can be used to attach it to the skin, and its effect lasts approximately 5 to 10 minutes. The expected total time of each treatment session will be 20 to 30 minutes. The treatment points were selected in a consensus process by professors and researchers who have an acupuncture and moxibustion specialist license in Korea based on a text book [24], literature reviews [12,14,25], and the points used in our previous pilot study. The moxibustion group will receive moxibustion treatment on the affected knee(s) at 6 standard acupuncture points (ST36, ST35, ST34, SP9, Ex-LE04, and SP10) plus up to 2 points of ‘ashi’, if needed, according to the determination of the practitioners (Figure 3). The moxibustion group will also receive the care provided to the usual care group.

### Usual care

According to pragmatic design, the control group will not use a placebo procedure. Both the moxibustion and usual care groups will be allowed to use any type of treatment desired, including surgery, conventional medication, physical treatment, acupuncture, herbal medicine, over-the-counter drugs, and other active treatments. However, the participants will be asked to inform the research investigators of any new treatments performed after entry into the trial. Educational materials explaining KOA and self-exercise will be provided to each group. The participants in both groups will perform self-exercise, which consists of 1) stretching the hamstrings, biceps femoris, and ankles on both sides 3 times per day and 2) muscle exercises for the hamstrings and biceps femoris (including isometric and isotonic exercises) on both sides according to the disease status during the trial period.

### Outcome

#### Primary outcome measurement

The primary outcome with respect to the effectiveness on KOA will be the mean change in the global scale value of the Western Ontario and McMaster Osteoarthritis Index (WOMAC) from baseline to 5 weeks. The WOMAC is one of the most widely used instruments and is composed of 24 questions to assess disability related to osteoarthritis. It includes 3 subscales to measure pain (5 questions), stiffness (2 questions), and physical function (17 questions) in KOA, and higher scores indicate more severe impairment [26,27]. There is a Korean version of the WOMAC (K-WOMAC), and Bae *et al.* confirmed its reliability, validity, and responsiveness [28]. We will therefore assess participants at the second visit (before starting moxibustion treatment) and at 5 weeks and 13 weeks from baseline using the K-WOMAC.

### Secondary outcome measurements

Secondary outcomes include the mean changes in the WOMAC subscales (pain, stiffness, and function) at 5 and 13 weeks from baseline. As such, we will investigate which subscales affect the global scale value of the WOMAC, which is the primary outcome measurement.

Health-related quality of life (HRQL) will be assessed by the Short-Form 36v2 Health Survey (SF-36) at 5 and 13 weeks from baseline. The SF-36 is used to measure the general health status and quality of life of patients and non-patients. It comprises a total of 36 questions in 8 categories, and higher scores indicate a better level of function. Han *et al.* developed a Korean version of the SF-36 and assessed its reliability and validity for use in health-related quality of life measurements for elderly Korean patients [29].

Depression will be assessed by a mean change in Beck Depression Inventory (BDI) scores at 5 and 13 weeks from baseline. The BDI, developed by Beck AT in 1967, is a 21-question multiple-choice questionnaire that is one of the most widely used scales for measuring the severity of depression. It is composed of items related to the depression symptoms of hopelessness and irritability, cognition (such as guilt and feelings of being punished), and physical symptoms (such as fatigue and weight loss); higher total scores indicate more severe depressive symptoms [30].

To assess physical activity, a physical performance test will be performed at 5 and 13 weeks from baseline. The physical performance test is composed of the timed stand, standing balance, and 6-minute walk tests. 1) The timed stand test evaluates lower extremity muscle power by measuring the time required to complete 10 full stands from a sitting position. 2) The standing balance test assesses balance in particular positions, such as tandem, semi-tandem, side-by side, and one-legged stands. 3) The 6-minute walk test measures the distance walked at the greatest speed possible within 6 minutes to assess the functional exercise capacity of patients [31].

Global knee pain will be assessed using the patient global assessment and numerical rating scale (NRS). The patient global assessment score is a self-reported 5-point measurement used to evaluate overall improvement after treatment. Participants individually evaluate their improvement from baseline by selecting one of 5 options (much improved, minimally improved, no change, minimally worse, or much worse) at 5 and 13 weeks from baseline. The pain NRS (0 = no pain and 10 = worst possible pain) is a standard instrument in chronic pain studies that is used to measure pain intensity [32]. We will evaluate the mean change in the NRS within 7 days of the study visits at 5 and 13 weeks from baseline.

The treatment expectancy of participants will be evaluated by the Treatment Expectancy Questionnaire (1 = not at all, 5 = somewhat and 9 = very much), which has been transformed and translated into Korean from the “Credibility and Expectancy Questionnaire” [33]. Participants will be asked “How much do you feel moxibustion therapy will help to reduce your symptoms?” at the first visit.

A blood sample will be obtained at baseline and 5 weeks from baseline to measure the C-reactive protein (CRP) levels and the erythrocyte sedimentation rate (ESR). We will therefore evaluate inflammation and tissue damage after moxibustion treatment. The detailed outcome measurement time points are provided in Table 1.

**Table 1:** Schedule for treatment and outcome measurements

	**Period**	**S**							**T**							**F**
	**Visit**	**1**	**2**	**3**	**4**	**5**	**6**	**7**	**8**	**9**	**10**	**11**	**12**	**13**	**14**	**15**
	**Week**		**1**	**1**	**1**	**2**	**2**	**2**	**3**	**3**	**3**	**4**	**4**	**4**	**5**	**13**
Informed Consent		●														
Demographic Characteristics		●														
Medical History		●														
Knee X-Ray		●														
Inclusion/Exclusion Criteria		●														
Conformity Assessment		●														
Treatment Expectancy Questionnaire		●														
Blood Test		●													●	
Vital Signs		●	●	○	○	○	○	○	○	○	○	○	○	○	●	●
Change in Medical History			●	○	○	○	○	○	○	○	○	○	○	○	●	●
Random Allocation			●													
Moxibustion Treatment			○	○	○	○	○	○	○	○	○	○	○	○		
K-WOMAC			●												●	●
Beck Depression Inventory			●												●	●
The Short-Form 36v2 Health Survey			●												●	●
Pain Numerical Rating Scale		●	●												●	●
Physical Function Test			●												●	●
Patient Global Assessment			●												●	●
Safety Assessment			●	○	○	○	○	○	○	○	○	○	○	○	●	●

*S*: Screening Period, *T*: Treatment Period, *F*: Follow-up Period, *K-WOMAC*: Korean Western Ontario and McMaster Osteoarthritis Index, ●: Both the moxibustion group and the usual care group, ○: Only the moxibustion group.

### Sample size

We calculated the sample size according to our previous pilot study. A 2-sided 5% significance level and 80% power were considered to detect a mean difference in the WOMAC of -6.65 with a standard deviation (SD) of 15.4. Approximately 84 participants in each group were calculated to be required. Assuming a dropout rate of 20%, the sample size will be n = 106 for each group (total n = 212). A total of 32 participants will be assigned at the Korea Institute of Oriental Medicine (Daejeon University Hospital), and the remaining 180 participants will be equally assigned at the other 3 study hospitals. To recruit this number of participants, a 6-month study period is anticipated based on our pilot study.

### Statistical analysis

The statistical analysis will be performed on the intention-to-treat basis with a 95% confidence interval using SAS (Version 9.1.3; SAS institute Inc., Cary, NC, USA). Missing data will be replaced according to the principle of the last observation carried forward (LOCF) method. The null hypothesis is that there will be no difference in the change in the primary outcome between the moxibustion group and the usual care group after 5 weeks of treatment. The baseline characteristics will be shown as the mean ± SD for continuous data (e.g., age and duration of disease) and n (%) for categorical data (e.g., gender and Kellgren/Lawrence Scale).

To analyse the baseline characteristics, we will perform a 2-sample *t*-test or a Wilcoxon rank sum test for continuous data and a chi-squared test or Fisher’s exact test for categorical data, according to whether the data are normally distributed. An analysis of covariance (ANCOVA) will be performed for the primary endpoint (global scale value of WOMAC) and secondary endpoints (WOMAC subscales (pain, stiffness, and function), HRQL, depression, physical activity, and patient global assessment) at 5 weeks and 13 weeks from baseline. The ANCOVA model will include group as a fixed effect and baseline measurements and centres as covariates. The mean difference from baseline values to the end of treatment in each group will be examined using a paired *t*-test or a Wilcoxon signed rank test. To identify a trend, we will also perform a repeated measures analysis of variance.

### Additional analyses

#### Economic evaluation

The economic evaluation will be conducted from a social perspective, and all costs and consequences of the competing interventions will be considered regardless of who pays for or benefits from them. All cost data will be collected through a cost questionnaire that participants will complete at 5 weeks and 13 weeks after baseline. Researchers will explain these to all of the participants, and obtain the consent before collecting economic data. The costs considered in the present analysis will include the direct healthcare-related costs of moxibustion treatment, physician visits, hospital stays (without consideration of private billings), and any drugs (including patient co-payments). In addition to health insurance costs, the indirect costs caused by lost workdays will also be taken into account. Health utility will be measured in quality-adjusted life years calculated for each patient using the SF-36 at 5 weeks and 13 weeks after baseline, and the K-WOMAC pain subscale will also be used to analyse cost-effectiveness. The cost-utility and cost-effectiveness analyses will be performed through a decision analysis. The analysis period will be 5 weeks, and the trend will also be evaluated at 13 weeks.

### Qualitative research

We will also conduct a qualitative study based on the grounded theory to elucidate the meaning of the experiences of moxibustion treatment for KOA. Sixteen KOA patients who will be selected by convenience sampling will be included in the qualitative study if they consent to give a recorded face-to-face interview. We will conduct individual in-depth interviews using open-ended questions at a site where they feel comfortable. The interviewer will ask the participants the key question, “Tell me about your experiences with obtain moxibustion treatment for treating KOA”, and additional questions, such as “What is the most difficult activity due to KOA?”, “Why did you choose moxibustion treatment? What was your motivation?”, “What was your expectation when you participated?”, “What types of changes did you feel after you received moxibustion? Were there any symptom changes after moxibustion treatment?”, “Did you have any difficulties or problems during the moxibustion treatment? What did you do to overcome these troubles?”, “Will you choose moxibustion treatment again when you have a similar or other symptom?”, and “Will you recommend moxibustion treatment to others?”. The data will be transcribed from the recording and analysed using the coding scheme, and researchers will then categorise them according to the process for qualitative research methods.

### Data and safety monitoring

Regular monitoring that will be clarified in a standard operating procedure will be conducted at each site to ensure good data quality. Monitors will evaluate whether the case report forms are properly written and whether the recruiting and treatment procedures are adequately performed according to the protocol. Investigators from each site will be contacted to discuss whether it is necessary to revise the study protocol or inclusion criteria and other important issues. The investigators and independent researchers will assess the progress of the clinical trial and severe adverse events and determine whether they are acceptable and whether it will be necessary for the trial to be modified or stopped.

### Adverse events

All unexpected and unintended responses that are not necessarily related to moxibustion treatment will be reported by the participants and practitioners at every visit. The adverse events known to occur following moxibustion treatment include blisters, redness, itching, burns, and respiratory symptoms. To avoid potential adverse events, the treatment day will be modulated within 3 days if the practitioner judges that the participant is not suitable for moxibustion treatment or the participant indicates moderate fatigue or an abnormal health condition.

### Participant protections and ethics

To protect human participants, the protocol was written according to general ethical guidelines, such as the Declaration of Helsinki and Korean Good Clinical Practice and was approved by the institutional review board of each trial centre (Daejeon University Hospital, Kyungwon University Gil Oriental Medical Hospital, Dongshin University Gwangju Oriental Hospital, and Semyung University Oriental Medicine Hospital). The Korea Institute of Oriental Medicine certified the ethics approval of the clinical centres as above. The study participant consent process includes information about potential risks, benefits, alternatives, and responsibilities during the trial. Before participants agree to participate in this trial, researchers will explain this information in detail in person.

## Discussion

Moxibustion and acupuncture have been widely used to treat the pain and functional limitations of KOA in Korea [20]. Some trials [11-14,34] have been conducted to evaluate the effectiveness of moxibustion in treating KOA in Korea and China. Moxibustion and NSAIDs were compared in 3 RCTs [11-13], combination therapy (moxibustion plus NSAIDs) and NSAIDs were compared in 1 RCT [14], and moxibustion and a hot pack were compared in 1 RCT [34]. According to these trials, single or combination moxibustion therapies increased the total response rate and decreased symptoms of pain in KOA patients. However, the processes of random sequence generation and allocation concealment were not reported in 4 trials [11,13,14,34]. Moreover, none of the trials used valid and reliable assessment tools, such as the WOMAC, and assessor blinding was not described. It is therefore not possible to exclude the possibility of a high risk of bias and determine the effectiveness of moxibustion treatment for KOA.

Concealment is the most critical factor of the allocation process, and a high risk of bias is generated by unconcealed allocation [35,36]. In a recent well-designed systematic review of acupuncture for chronic pain, only RCTs that used an unambiguous method of allocation concealment were included [37]. Therefore, we designed our protocol with adequate methodology of random sequence generation and allocation concealment using validated assessment tools, such as the WOMAC, SF-36, and BDI, and monitors will regularly evaluate whether the trial is being conducted according to the planned protocol.

Mixed-method research is the most recent systematic approach of using 2 or more research methods to answer a single research question. This approach has the advantages of expanding the understanding of the procedure's effects and verifying results from another perspective compared to an approach using a single method because the supplemental component enhances the validity of the study [36,38]. This method has been used to determine the effectiveness of interventions in recent studies on complementary and alternative medicine [39-41]. To comprehensively understand the effectiveness of moxibustion for KOA patients, we will conduct not only a quantitative data analysis but also economic and qualitative data analyses. A nested economic evaluation and qualitative research within a pragmatic clinical trial will be used as a mixed-methods approach to answer the question from various perspective on the reasons for using moxibustion for KOA.

One limitation of this study is the possibility of a high risk of bias regarding blinding because we will use usual care as a control instead of a sham procedure. One study [42] on functional constipation used sham moxibustion to blind participants to evaluate the efficacy of moxibustion, but a blinding test was not performed, and it is necessary to cover the eyes with a patch to successfully blind participants to the sham moxibustion device [43]. The researchers who designed this protocol agreed that it would be difficult to blind participants using any previously developed sham device [17,43,44], and that a sham device is difficult to use because of the high prevalence of moxibustion in Korea and there are technical challenges associated with developing a sham device. Additionally, it is more appropriate to use a pragmatic design to answer our research question, which is whether moxibustion treatment is effective in treating KOA in real practice. Therefore, we used the pragmatic approach of comparing treatment to usual care, permitting additional treatment points as well as standard treatment points, and using patient-centred outcomes in multiple centres.

This pragmatic randomised controlled trial using the mixed-methods approach may provide rigorous evidence for the effectiveness of moxibustion in treating KOA.

## Competing interests

The authors declare that they have no competing interests.

## Authors’ contributions

SL wrote the study protocol and drafted this manuscript. KHK made a substantial contribution to designing the study protocol. THK participated in the study design of the qualitative study and in critical revision. JEK, JHK, and JWK participated in the critical revision of the manuscript. KWK participated in the design of the statistical analysis and the cost-effectiveness analysis. SYJ, ARK, HJP, and MSS participated in the design of the outcome measurements and assessing the outcomes. KEH, HSS, JBC, and HJK helped to draft the manuscript. SMC had final responsibility for the decision to submit for publication. All of the authors read and approved the final manuscript.

## Pre-publication history

The pre-publication history for this paper can be accessed here:

http://www.biomedcentral.com/1472-6882/13/59/prepub
